# Editorial

**Published:** 1880-06

**Authors:** 


### Editorial.

At a meeting of the Chicago Gynæcological Society, held Dec. 27, 1878, the subject of placenta previa being under discussion, Dr. Edward Warren Sawyer called attention to a peculiarity of the uterine tissue at the placental site which had not before been observed. THE JOURNAL AND EXAMINER, for Feb., 1879, page 175, contains a report of these remarks. The speaker said: "Another point is one which I have never seen alluded to in the books, namely, the friability of the uterine tissue upon which the placenta is developed." After speaking of two fatal cases which he thought due to a tear of this friable area, he said: "In the light of this experience, I would in future cases, use extreme care in all procedures which would stretch the uterine tissue in the cervical zone, upon which the placenta is attached."

The *London Lancet*, for Oct. 25, 1879, contains an article by Dr. George Roper, of London, in which the writer reports the same observation, but made independently of Dr. Sawyer.

*The Boston Medical and Surgical Journal*, Jan. 1, 1880, alludes to this important observation of Dr. Roper, but seems ignorant of the fact that Dr. Sawyer ante-dated Dr. Roper by nearly a year. In a recent letter from the latter to Dr. Sawyer, the writer says: "You have clearly hit on the same observation as myself;" and further: "I am by no means ambitious as to the originality of this observation, and as I shall probably write an article on placenta previa, when I have time, I shall have much pleasure in referring to your own observations."

The London gentleman having thus politely admitted the priority of Dr. Sawyer, we feel it to be just to call attention to the subject in this manner.

